# Recent Hits Acquired by BLAST (ReHAB): A tool to identify new hits in sequence similarity searches

**DOI:** 10.1186/1471-2105-6-23

**Published:** 2005-02-08

**Authors:** Joe Whitney, David J Esteban, Chris Upton

**Affiliations:** 1 Department of Microbiology and Biochemistry, University of Victoria, Victoria, BC, V8W 3P6, Canada

## Abstract

**Background:**

Sequence similarity searching is a powerful tool to help develop hypotheses in the quest to assign functional, structural and evolutionary information to DNA and protein sequences. As sequence databases continue to grow exponentially, it becomes increasingly important to repeat searches at frequent intervals, and similarity searches retrieve larger and larger sets of results. New and potentially significant results may be buried in a long list of previously obtained sequence hits from past searches.

**Results:**

ReHAB (Recent Hits Acquired from BLAST) is a tool for finding new protein hits in repeated PSI-BLAST searches. ReHAB compares results from PSI-BLAST searches performed with two versions of a protein sequence database and highlights hits that are present only in the updated database. Results are presented in an easily comprehended table, or in a BLAST-like report, using colors to highlight the new hits. ReHAB is designed to handle large numbers of query sequences, such as whole genomes or sets of genomes. Advanced computer skills are not needed to use ReHAB; the graphics interface is simple to use and was designed with the bench biologist in mind.

**Conclusions:**

This software greatly simplifies the problem of evaluating the output of large numbers of protein database searches.

## Background

Advances in technology have increased the speed and reduced the cost of DNA sequencing. This has resulted in a dramatic increase in the number of sequences contributed by both large sequencing centres and individual laboratories to sequence databases. Public biological sequence databases are growing at an ever-increasing rate, with 9 million new sequences being added to GenBank from August 2002 to August 2003 alone [1]. Currently, the GenBank database has almost 42 billion nucleotides from over 32 million sequences. The number of whole genome sequences of eukaryotes, prokaryotes and viruses is also increasing rapidly. Accordingly, tools like NCBI BLAST, which search those databases for sequences similar to a given query sequence, return larger and larger sets of results.

Sequence similarity searching is a powerful tool to help develop testable hypotheses in the quest to characterize genes and other DNA sequences and infer structural, functional or evolutionary relationships. Researchers interested in identifying new matches to query sequences, which may be a few genes or even whole genomes, must search through massive amounts of alignment data to retrieve new and interesting matches. In order to keep up with the growing databases, the researcher must submit the same queries periodically. However, the new results, no matter how significant, are often buried in a long list of results that were previously obtained on past searches.

ReHAB (Recent Hits Acquired from BLAST) is a new software package that was developed to address these problems. ReHAB performs PSI-BLAST [2] searches of a protein sequence database and keeps a database of all significant alignments ("*hits*") obtained; these searches are performed on a regular schedule against updated versions of the sequence database. It then compares the sequences in the new PSI-BLAST result with the ReHAB *hits *database to identify new matches resulting from recently deposited sequences. The complete ReHAB *hits *database can then be queried by date using a simple GUI to allow the researcher to easily identify new *hits*; these are highlighted, and pairwise or multiple alignments can be performed to assess the quality of the match. As well as filtering out results that have been found previously, the ReHAB browser can filter out hits against sequences that are identical to the sequences being submitted as queries (such as orthologs of the query sequence).

ReHAB is designed to be a practical tool for searching NCBI database updates with large numbers of query sequences. For example, our laboratory uses it with all open reading frames (ORFs) from fully sequenced poxvirus genomes (over 7000 query sequences). As the number of sequenced virus genomes continues to increase, the number of hypothetical ORFs of unknown function also expands. This is particularly true for large viruses like poxviruses, baculoviruses, and herpesviruses that possess many virulence genes that are not part of the core set of genes that define a virus family [3]. There are also numerous core genes for which no known function has yet been identified; for example, of 49 completely conserved protein families in poxviruses, there are 11 with completely unknown function and at least 5 others with only poorly defined function.

Other programs have been previously created to deal with this particular issue, including DBWatcher [4], SEALS [5], Swiss-Shop [6], Sequence Alerting System [7] and BLAST Search Updater [8]. However, WWW-based programs are not well suited to searching with large numbers of query sequences, and there may be concerns with a shut-down of service (as occurred with Sequence Alerting System) or allowing proprietary data out of a secure network. Other programs may be complicated to use, or require users to directly interact with UNIX operating systems. ReHAB is specifically designed for searching with large numbers of query sequences and can support a number of research groups; it also provides a user-friendly graphical interface. The client will run on most major operating systems including Mac OS X, Windows, Linux and Solaris.

## Implementation

### Design rationale

ReHAB was implemented for the Java platform to simplify the support of multiple operating systems including Linux, Microsoft Windows, Solaris, and Mac OS X. Users initially access and launch the application (client) from a web page using Java Web Start, which also automatically downloads updated versions as they become available. This ensures users are taking advantage of improvements or added features in the latest software version. Furthermore, coding in Java allows interoperability with existing applications developed in our laboratory, including Base-By-Base [9]. Our choice was reinforced by past successes with the Java platform and Java Web Start for implementation and distribution of programs such as VOCs [10], VGO [11], and Base-By-Base [9].

### Components

**Figure 1:**
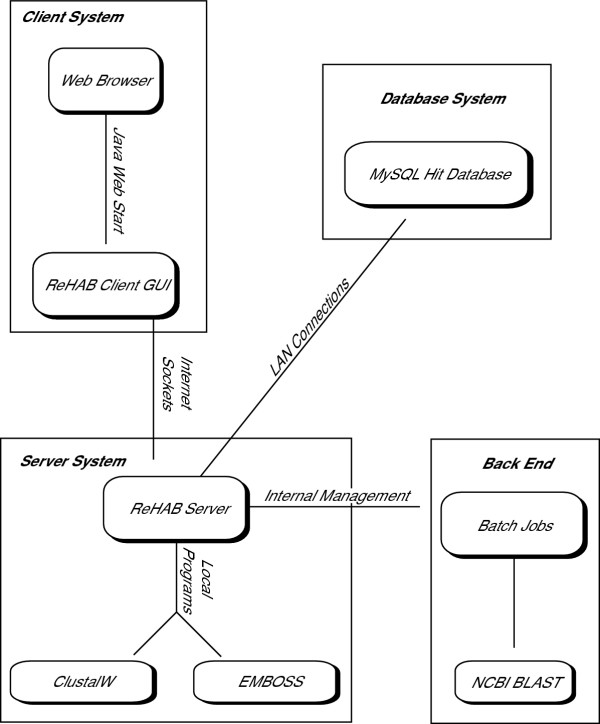
**Organization of ReHAB processing steps. **ReHAB is set up as four main components: the client, the *Hits *Database, the server and the back end. See text for details.

ReHAB consists of four main components (Figure 1): (1) a MySQL relational database that stores information about *hits*, including biological sequences, alignments between them, and other categorization and annotation data; (2) a Java server that provides access to programs which cannot be run locally by the client on arbitrary user workstations, such as NCBI BLAST and EMBOSS [12] utilities; (3) a Java Swing graphical client, downloaded and launched on client machines using Java Web Start; (4) and a back-end Java program which runs alignment programs and compiles results in the database. Each of these components is described in more detail below. Although all components can be run on a single machine, it is envisioned that a single server will support a variety of users dispersed on an intranet or the Internet; if required, it is simple to offload the batch database searching to a more powerful cluster or grid system.

### *Hits *database

There are four types of information stored in the ReHAB database: (1) biological sequences and their annotations, both those used as queries in BLAST searches and those which have been returned as *hit *subjects; (2) information on each query/subject pair (*hit*), gathered from individual search results and alignment programs (including bit-score, date entered, and percent identity); (3) organizing information, such as which query sequences belong to which organisms; and (4) other caching information, used to speed performance of server-side program functions. To reduce the amount of required storage space, actual alignments are not stored, but are regenerated for presentation when the user selects the specific query sequence or query-target pair to be viewed. Query sequences, which are entered using a simple FASTA-like format that includes additional annotation information in the identifying line, need only be submitted once to ReHAB since they are stored for future search cycles.

### Back-end processing

The work of running PSI-BLAST searches is done in batch mode by the NCBI blastpgp program against a local copy of the NCBI non-redundant (NR) protein database. PSI-BLAST is performed for three iterations without filtering procedures (such as for low-complexity regions). Hits with an E-value less than 0.001 are used to generate the scoring matrix for the subsequent cycle. To increase speed, searches with query sequences that result in no new hits are terminated after the first cycle. Those with new hits scoring below the threshold continue to the third cycle. PSI-BLAST was chosen because it is a more sensitive search method than BLASTP. The searches do not need to be performed on the same machine on which the database or server components are installed. XML output from blastpgp is parsed and relevant information about each hit is stored in the ReHAB database. In addition to scores and identifying information, target sequences are copied into the ReHAB database to ensure that they are available for analysis in the future, even if they are no longer available from NCBI. This is important because, although NCBI does not actually remove sequences from its database, it may change the identifier of a sequence if it is corrected, updated, or merged with another identical entry. Any changes to a pre-existing entry are added to the database, but it is not registered as a new hit.

### Server

The server component consists of Java RMI classes that provide remote access to local facilities, and a loading program that registers those classes with an RMI Registry installed on the server from which the client will be downloaded. Requirement for the server is a system that can support Java 1.4.1 and MySQL 4.0.

### Client GUI

The Java Swing client component allows a user to browse the information collected in the database by the back-end program. When the client is downloaded and launched from a website, it connects to the server and database specified in its configuration file.

The client program visually presents summary information about hits added to the database, and allows the user to quickly locate new, relevant hits and the sequences involved. There are five main views available in the client: (1) The management console lists the available databases, and has options for creating new databases or adding files to existing databases, (2) The Hits Browser window lists the organisms for which query sequences have been added in the database, and allows users to select filtering and highlighting options, (3) a Hits Summary, which displays the results in a table with highlighting to mark new hits, and (4) an HTML output or (5) a Hits Manager that displays detailed information about retrieved sequences and alignments.

## Results and discussion

### Finding new hits

**Figure 2:**
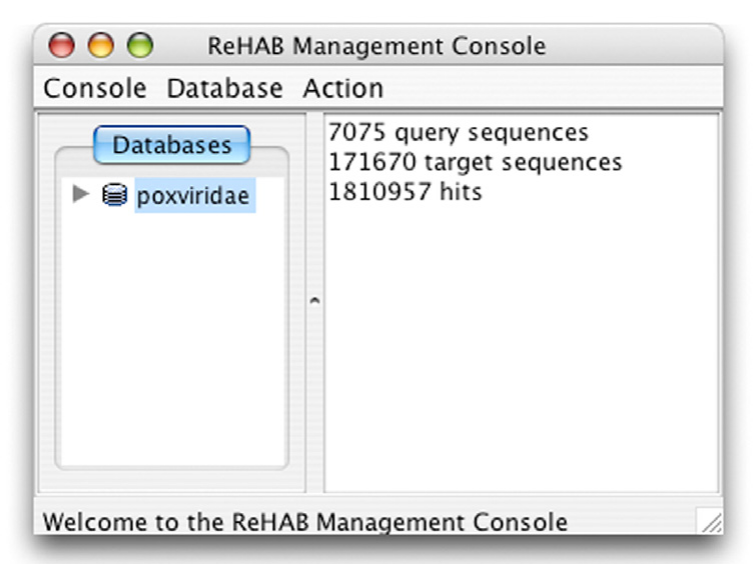
**ReHAB management console. **A database is selected from the list on the left, and statistics are displayed on the right. More hits than target sequences are displayed because query sequences can match multiple targets. Double-clicking on the database or selecting an option from the Action menu allows users to browse the selected database.

**Figure 3:**
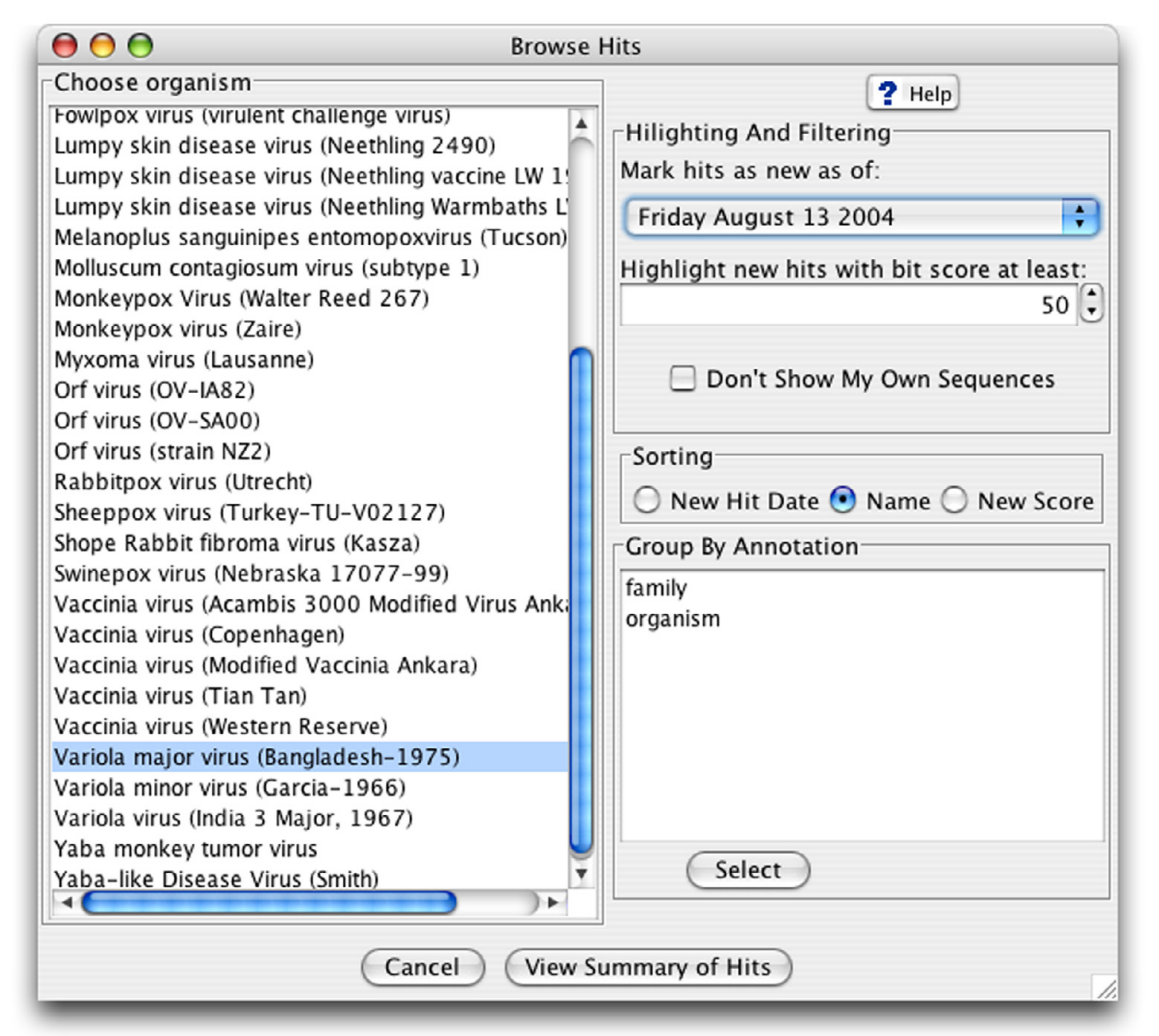
**Hits browser window. **The ReHAB database is searched by selecting an organism name, then choosing the desired highlighting and filtering options. Clicking on "*Show Summary" *opens a new window to display the results.

**Figure 4:**
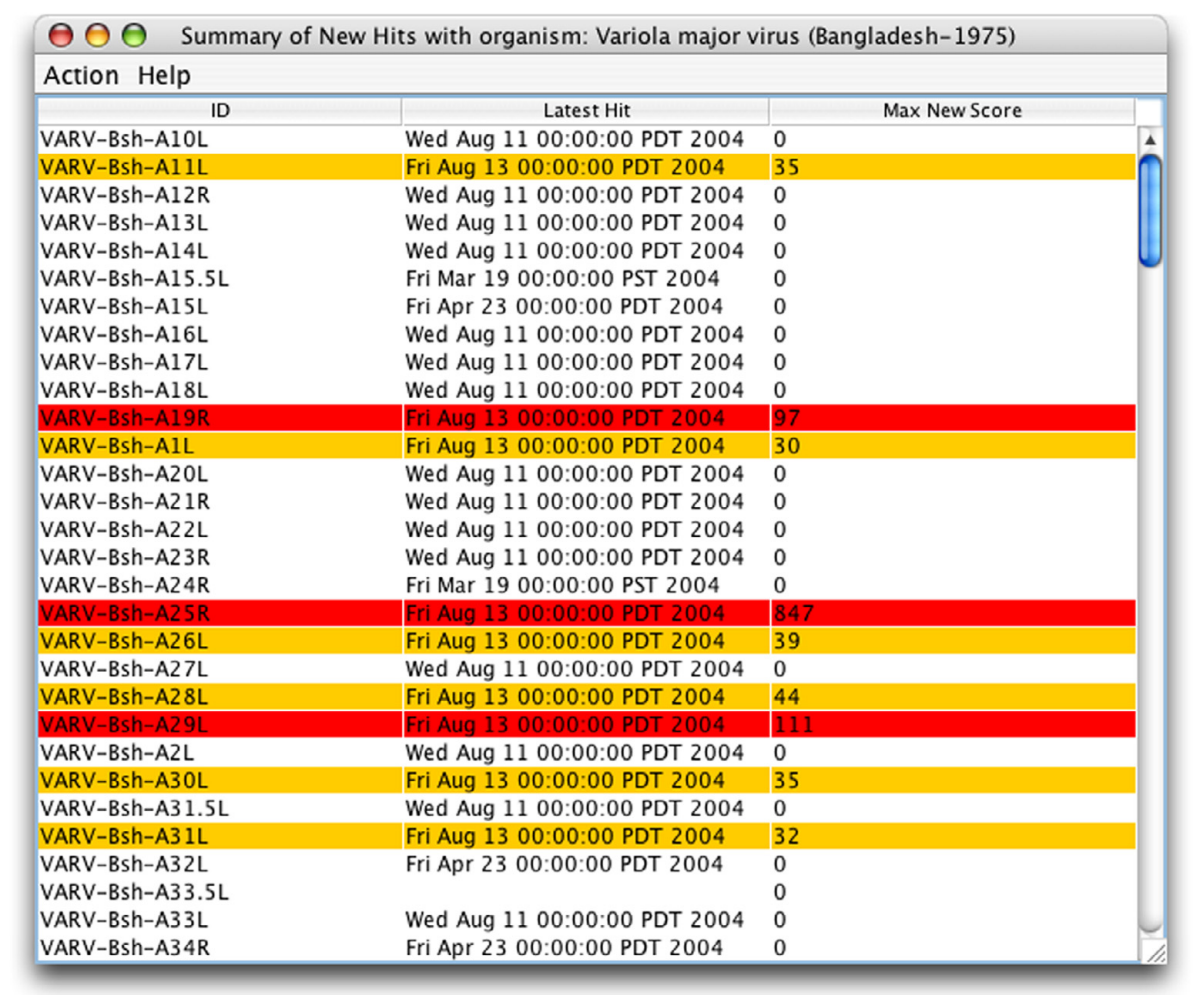
**Query sequences with new hits are highlighted. **A user defined threshold (in the Browser window) is used to define the minimum bit-score that is highlighted in red, and all new hits with lower scores are highlighted in yellow. The *Latest Hit *column indicates the date of the most recent hit. Those with no entry in this column have no hits in the database (for example, VARV-Bsh-A33.5L). Sorting of the entries can be changed by clicking on the column heading. Details about the hits can be obtained by right-clicking on the entry or selecting an option in the Action menu.

ReHAB is a tool that works with BLAST to identify new hits in updated versions of sequence databases. It allows the researcher to ask the question: "*what new sequences match my sequences since the last time I searched?" *In the example of our work, the query sequences are all the ORFs of the fully sequenced poxvirus genomes (36 genomes, 7075 query sequences). These sequences are used to query the NR NCBI protein database and a MySQL database of all *hits *is generated and stored (Figure 2). Databases of *hits *for other virus families maintained by The Virus Bioinformatics Resource (TVBR; herpesviruses, baculoviruses, coronaviruses, and adenoviruses) will also be available in the near future. The *hits *database can then be accessed by double-clicking on the database name or by selecting "*browse by organism*" in the Action menu. This opens a new window to present browsing, sorting, and highlighting options (Figure 3). The user can browse by organism name, such as "*Variola Virus strain Bangladesh-1975*". To highlight recent *hits*, the "*date option*" is chosen to define the date after which the *hits *are considered new. The available dates are those on which the query sequences were searched against a then current NR NCBI database. The output can be sorted based on three criteria (name, new hit date, or maximum new hit bit-score) by selecting the appropriate radio button. The results are presented in a new window, using colors to indicate new hits (Figure 4).

Since all new *hits *are not necessarily significant, results are highlighted in different colors depending on the bit-score. The user can change the default threshold of the minimum bit-score, to show new *hits *scoring above this cut-off in red and new *hits *scoring below it in yellow. Since all query sequences that have new *hits *are highlighted, any that remain unhighlighted do not have new *hits*. However, unhighlighted queries may have significant *hits *from previous searches. The "*Latest Hit*" column indicates this fact: query sequences showing *hits *only from previous searches show an older hit date, and a bit-score of "0" in the "*New Hit Score*" column. Unhighlighted sequences with no information in the "*Latest Hit*" column do not have any hits in the database or they have been filtered out (see below).

**Figure 5:**
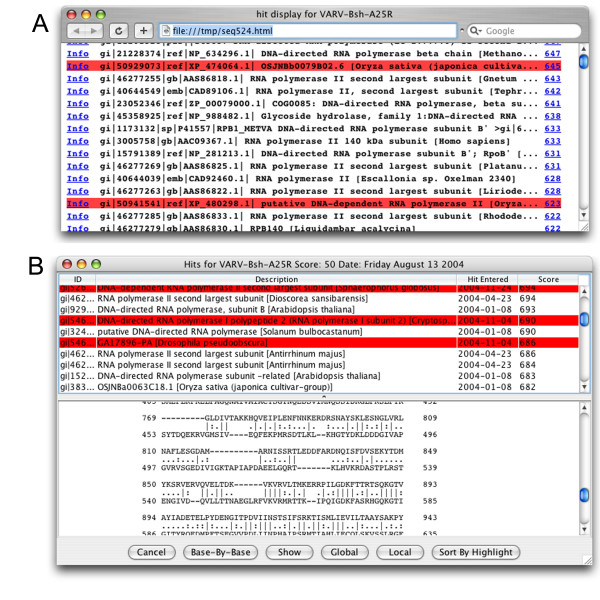
**Analysis of hits. **Hits can be viewed in A) HTML output, showing all hits listed in order of descending score, followed by a pairwise Needle alignment of the query and target sequence. The *Info *hyperlink links to the NCBI entry for the target sequence, and the *score *hyperlink takes the user to the Needle alignment. B) The Hits Manger window, which allows the user to sort hits and view pairwise or multiple alignments, or view selected sequences in FASTA format. A global alignment is shown between the query sequence and the top scoring new hit.

Information about the *hits *can be viewed in two ways. Selecting "*HTML Report*" launches the user's default web browser and displays the "*hit-list*" in familiar BLAST-style (Figure 5a). *Hits *are displayed in descending order of bit-score, however, a key feature of this program is that new hits are highlighted in red or yellow. The pairwise alignment can be displayed rapidly by clicking on the score (a hyperlink). In contrast to the usual BLAST output, which presents the local alignment found by BLAST, a global alignment produced by Needle [12,13] is shown. More information can be obtained about the target sequence by clicking on the link to the NCBI file for that entry. Alternatively, a full list of hits can be viewed in the "*Hits Manager" *window (Figure 5b). Here, sequences can be sorted by highlight, and pairwise or multiple alignments can be performed. Pairwise alignments are displayed by selecting a single target sequence and clicking the "*Global*" button for global alignments produced by the EMBOSS program Needle or the "*Local*" button for local alignments generated by the EMBOSS program Water [12,14]. Multiple alignments are generated by selecting more than one target sequence and clicking the "*Base-By-Base*" button; the software automatically retrieves the appropriate sequences from the ReHAB *hits *database, performs a ClustalW alignment and passes the resulting multiple alignment to Base-By-Base, which functions as an alignment viewer and editor [9]. Finally, sequences in the *Hits Manager *can be viewed in FASTA format by clicking the "*Show*" button and can be copied from this window using standard keystrokes.

### Filtering out identical sequences

Unless a sequence has not been deposited in the public database, a sequence similarity search will return results including the query sequence itself, as well as nearly identical sequences that are orthologs of the query. ReHAB can block the highlighting of *hit *sequences that are also present in the query database when the "*Don't Show My Own Sequences*" option is selected; such sequences will not be shown or highlighted in the *Hits Results *window. However, these sequences and their alignments with the query can still be visualized in the *HTML Report *and *Hits Manager *windows, thus allowing the user to access all the available information. This feature becomes essential when new poxvirus genomes are added to the public database, since a large fraction of the queries will hit proteins in the new genome and signal a "*new hit*" report when there may be no other new hits in the database. Although these are clearly high scoring matches, they are expected and therefore must be masked in the analysis if the full value of ReHAB is to be realized.

### Browsing by other criteria

In the *browser *window (Figure 3), the user can chose to browse by the annotation included in each sequence's information line. In the case of our poxvirus sequences, useful annotations are organism name and protein family (as determined in POCs [10]). Selecting an item from the "*Group by Annotation*" list loads the new category in the list on the left side of the window. This sorting allows the user to quickly find query sequences of particular interest. For example, one may be interested in looking at only sequences from the *Ankyrin *family. Results can then be viewed and analyzed as described above.

### Setting up ReHAB with user selected sequences

**Figure 6:**
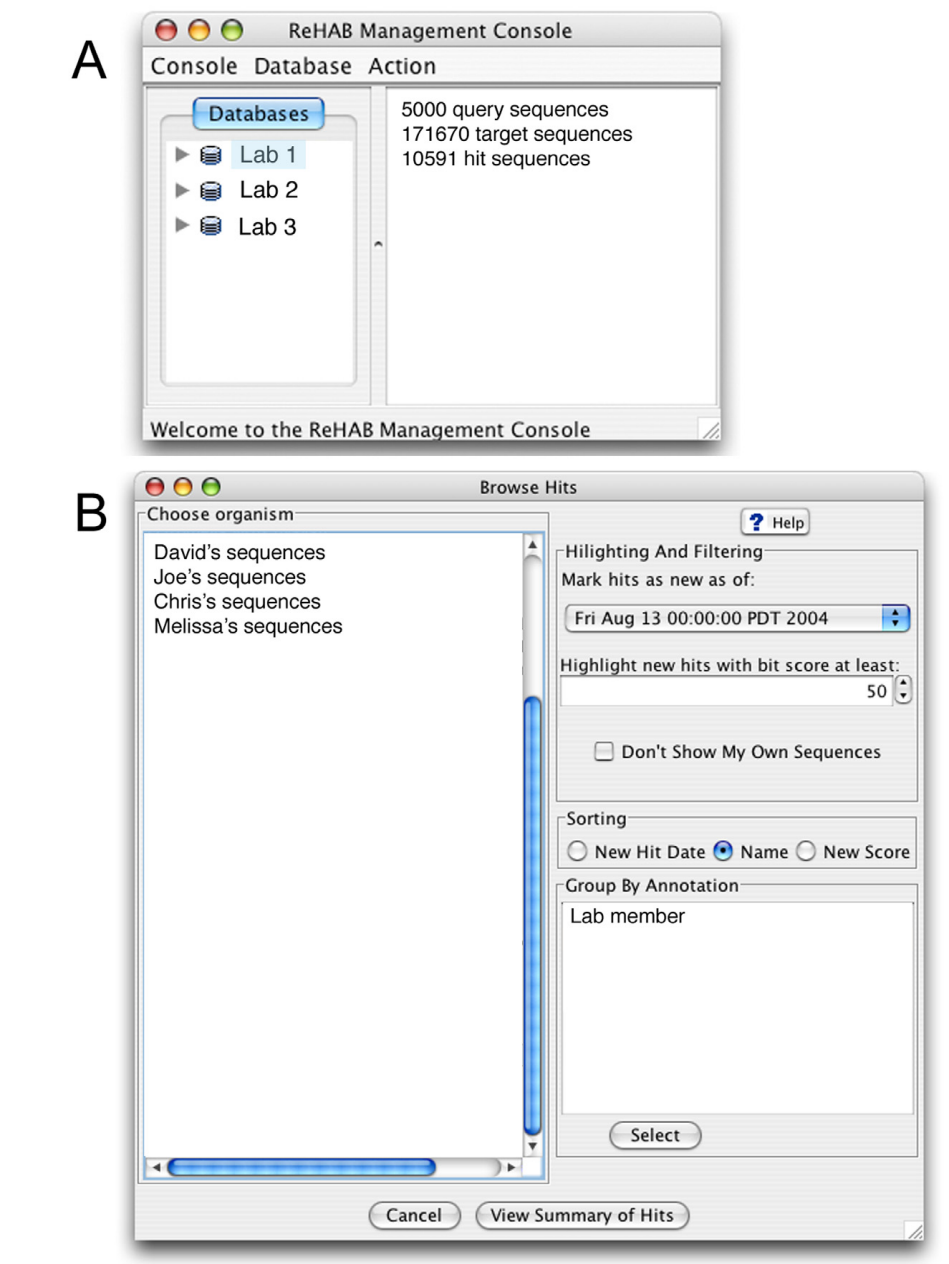
**ReHAB set up for other users. **A) Different laboratories in a department could have different query databases, which can be accessed as described in the text. B) The sequences within a lab's database could be annotated with individual lab member's names, or other identifying information, permitting individuals to view results for their own sequences of interest. In this way, large numbers of sequences of interest to a lab can be run simultaneously and frequently, and individuals can then browse results.

Researchers can use ReHAB to search databases with their own set of query sequences. In the example of our research, it is most practical to organize the query sequences by organism and protein family. Other researchers, however, may find other naming schemes to be more useful; no changes to the program or database are required. For example, a research group could organize *query sequences *and the *hits results *databases by laboratory name, and browsing of results could be by the researcher's name (Figure 6). Individual laboratory members would add query sequences to the database including their name in the identifying information line. In this example, the *laboratory name *would replace *virus family*, and *user names *would replace *organism names*. All query sequences would be searched in the same batch process, and each individual could then browse their sequences of interest. Users interested in establishing their own ReHAB database should contact the authors for assistance.

## Conclusions

The goal of this project was to build a software package to aid in the identification of new results returned from sequence similarity searches. To this end, we developed ReHAB, a tool that highlights new *hits *by comparing results from previously run searches to those with a recently updated database. ReHAB allows researchers to query the NR protein database with large numbers of sequences and can highlight, sort, and analyze results in a user-friendly graphical interface. It can also be used to rapidly create multiple alignments with any set of sequences returned by a BLAST search. This enables researchers to recognize new significant sequence matches in the mass of results generated by high throughput database search protocols.

## Availability and requirements

**Project name: **ReHAB

**Project home page: **

**Operating systems: **All platforms supporting Sun's JRE version 1.4.1 or compatible

**Programming languages: **Java, SQL

**Other requirements: **Java 1.4 or higher

**License: **GNU General Public License

**Restrictions for non-academic use: **Contact corresponding Author

## Authors' contributions

CU described and specified the features of and problems to be solved by ReHAB, tested the program and provided usage examples. JW implemented the software, both the Java components and the database schemata used to store alignment results. DJE tested the program and provided usage examples. All authors contributed to writing of the manuscript.

## References

[B1] Benson DA, Karsch-Mizrachi I, Lipman DJ, Ostell J, Wheeler DL (2004). GenBank: update. Nucleic Acids Res.

[B2] Altschul SF, Madden TL, Schaffer AA, Zhang J, Zhang Z, Miller W, Lipman DJ (1997). Gapped BLAST and PSI-BLAST: a new generation of protein database search programs. Nucleic Acids Res.

[B3] Upton C, Slack S, Hunter AL, Ehlers A, Roper RL (2003). Poxvirus orthologous clusters: toward defining the minimum essential poxvirus genome. J Virol.

[B4] DBWatcher. http://www-igbmc.u-strasbg.fr/BioInfo/LocalDoc/DBWatcher/.

[B5] SEALS. http://www.ncbi.nlm.nih.gov/CBBresearch/Walker/SEALS/index.html.

[B6] Swiss-Shop. http://www.expasy.org/swiss-shop/.

[B7] Sequence Alerting Systemm. http://www.bork.embl-heidelberg.de/Alerting/.

[B8] Boone M, Upton C (2000). BLAST Search Updater: a notification system for new database matches. Bioinformatics.

[B9] Brodie R, Smith AJ, Roper RL, Tcherepanov V, Upton C (2004). Base-By-Base: Single nucleotide-level analysis of whole viral genome alignments. BMC Bioinformatics.

[B10] Ehlers A, Osborne J, Slack S, Roper RL, Upton C (2002). Poxvirus Orthologous Clusters (POCs). Bioinformatics.

[B11] Upton C, Hogg D, Perrin D, Boone M, Harris NL (2000). Viral genome organizer: a system for analyzing complete viral genomes. Virus Res.

[B12] Rice P, Longden I, Bleasby A (2000). EMBOSS: The European Molecular Biology Software Suite. Trends Genet.

[B13] Needleman SB, Wunsch CD (1970). A general method applicable to the search for similarities in the amino acid sequences of two proteins. J Mol Biol.

[B14] Smith TF, Waterman MS (1981). Identification of common molecular subsequences. J Mol Biol.

